# The Relationship between Maternal Smoking and Infant Birth Weight: Improving Accuracy through Urine Cotinine Analysis and Effective Medical Record Strategies

**DOI:** 10.3390/children11081028

**Published:** 2024-08-22

**Authors:** Danica Vojisavljevic, Donna Rudd, Roger Smith, Yogavijayan Kandasamy

**Affiliations:** 1 College of Public Health, Medical and Veterinary Science, James Cook University, 1 James Cook Drive, Townsville, QLD 4814, Australia; danica.vojisavljevic@jcu.edu.au (D.V.); donna.rudd@jcu.edu.au (D.R.); 2 School of Medicine and Public Health, University of Newcastle, Newcastle, NSW 2308, Australia; roger.smith@newcastle.edu.au; 3 Department of Neonatology, Townsville University Hospital, 100 Angus Smith Dr, Townsville, QLD 4814, Australia

**Keywords:** cotinine, birth weight, sex, pregnancy, nicotine

## Abstract

Objective: We conducted a study to determine if antenatally collected maternal urine cotinine (a metabolite of nicotine) measurements can be used to assess the neonatal impact of nicotine exposure during pregnancy. This was a prospective longitudinal cohort of mother–infant dyads. Only term singleton pregnancies were included. The primary outcome measure was the correlation between maternal urine cotinine and infant birth weight. Methods: We analysed data from 238 mother–neonate dyads. Smoking habits were recorded during routine prenatal check-ups and urine samples were collected to measure cotinine and creatinine levels. Results: Urine cotinine was detected in 50.4% (120/238) of women from the whole cohort, but only 16% (38/238) self-reported as smokers (chi-square 39.7, *p* < 0.0001), and these women had significantly smaller babies (*p* = 0.010). There was a significant negative correlation between maternal urine cotinine and birth weight (Spearman’s coefficient = −0.0226, *p* = 0.013). Female babies born to women with nicotine in their urine were significantly smaller (*p* = 0.001). Conclusions: Infant birth weight significantly reduced in mothers with exposure to nicotine during pregnancy. The number of women exposed to nicotine during late pregnancy (measured in urine) was markedly higher than self-reported and national smoking percentages, suggesting an urgent need for an improvement in medical record reporting on smoking habits to better assess neonatal outcomes.

## 1. Introduction

Exposure to toxins and drugs during pregnancy creates suboptimal conditions for fetal growth and development [1,2,3]. It is well known that maternal cigarette smoking during pregnancy is one of the leading preventable causes of birth defects and impaired fetal development [4]. Despite this information, in Australia in 2021 [5], 8.3% of mothers reported smoking during the first 20 weeks of pregnancy, and of these, a substantial number of women (74%) reported smoking during the last 20 weeks of pregnancy. From 2011 to 2021, the number of women smoking during the first 20 weeks of pregnancy has slowly declined nationally (from 12.9%); however, the majority (70–74%) continued to smoke during the last 20 weeks of pregnancy [5]. Encouragingly, the proportion of Australian First Nations mothers who smoked during pregnancy declined by 9.2% between 2011 and 2020 [6]. However, a concerning number of pregnant women continue to smoke in regional and remote regions (ranging from 13.3–35.5%) [5].

Nicotine is the main addictive teratogen within cigarettes, and has been linked to adverse pregnancy outcomes and fetal development [7]. Nicotine causes vasoconstriction of uteroplacental blood vessels, crosses the placental barrier to enter fetus’ circulation, and accumulates in fetal tissues [4,8]. These mechanisms, directly and indirectly, impact pregnancy outcomes. Vasoconstriction of uteroplacental blood vessels reduces blood flow to the placenta, reducing blood flow to the fetus and reducing micronutrient and oxygen delivery [4]. Aoyagi et al. [9] found that, in animal models, the groups exposed to nicotine prenatally demonstrated a reduction in blood supply to all fetal organs compared with the control group. This reduction in blood flow can potentially cause fetal tissue hypoxia, fetal growth restriction and adverse postnatal health effects [9].

Additionally, nicotine is associated with other placental changes contributing to placental dysfunction. These alterations include the thickening of the trophoblastic membrane, increased collagen content in the villous stroma, decreased angiogenesis, the inhibition of trophoblast interstitial invasion, increased placental hypoxia and oxidative stress, and the downregulation of labyrinth vascularisation [10]. As the placenta functions as both a metabolic and endocrine organ, creating an effective maternal–fetal barrier, any disruption in placental function can potentially disrupt fetal growth and development [7,10]. Furthermore, the nicotine concentration in fetal circulation is much greater than in maternal circulation. In adults, 70–80% of circulating nicotine is metabolised through the liver; however, in utero, the fetal liver is underdeveloped and does not express the appropriate enzymes to allow nicotine metabolism [4,7].

The impact of smoking on neonatal birth weight is well recognised. Kataoka et al. [11] showed that, in full-term infants, birth weight decreased as the number of cigarettes per day increased, with a significant weight reduction in the category of mothers consuming 6 to 10 cigarettes per day. Similarly, a large meta-analysis by Kramer [12] highlighted a strong relationship between an increasing number of cigarettes smoked per day and reduced birth weight, which remained even when controlling for nutritional differences. Provision of smoking history to a health professional during antenatal visits and pregnancy continues to be suboptimal in most healthcare settings [13]. It is often obtained as part of a broader consult with health professionals where other medical histories pertinent to the pregnancy are obtained. An important consideration is that the accuracy of the data on smoking and the number of cigarettes smoked per day during pregnancy may vary during the pregnancy period. Indeed, Kramer [12] found smoking during the third trimester to have the most significant adverse effects on infant birth weight.

Cotinine, the major metabolite of nicotine, has become the biomarker of choice for assessing exposure to tobacco smoke (smoking) or environmental tobacco smoke (passive smoking) more accurately [14,15]. It has a long biological half-life of between 19 and 40 h in the body compared with nicotine, which has a short half-life of about 30 min to 2 h [14]. Serum cotinine levels, measured using ELIZA assays, have been shown to reflect dosage, showing a good correlation between the levels found in all biological fluids, urine, saliva, and cord blood. Considered less invasive, urinary cotinine levels are the most widely used marker of tobacco use and show high sensitivity [14].

We carried out a study to determine if maternal urine cotinine measurements can be used to assess the impact of exposure to nicotine during pregnancy on infant birth weight. The primary outcome measure was infant birth weight, and a secondary outcome measured any differences between male and female neonates. We hypothesised that maternal urine cotinine levels negatively impact birth weight, affecting both male and female neonates equally.

## 2. Materials and Methods

### 2.1. Study Design and Population

**Figure 1:**
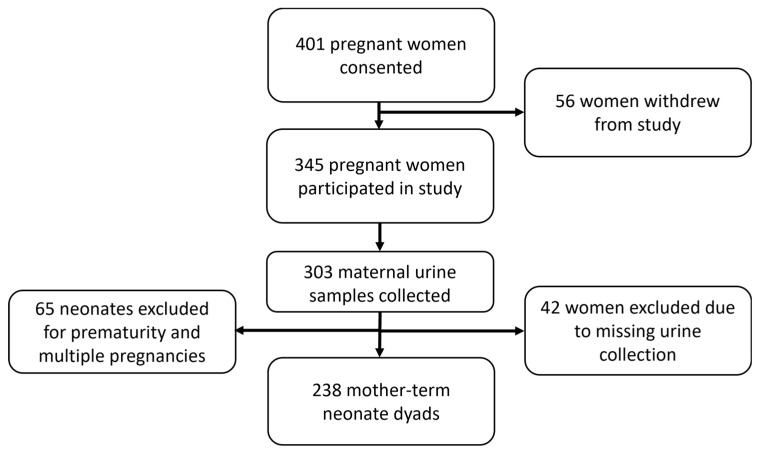
Flow diagram of participant recruitment.

The data presented in this manuscript are from “The Relationship between Maternal Health and Infant Kidney Development and Function” study, which was a prospective, longitudinal cohort of mother–infant dyads from pregnancy up until the child was two years of age carried out at the Townsville University Hospital (TUH), Queensland, Australia. TUH serves a region with a population of over 240,000 and 10,000 births annually [16]. This study was conducted between August 2019 and August 2021. All pregnant women presenting to the hospital during recruitment were invited to participate. A total of 401 women consented to the study. Of these, 56 women withdrew from the study, and 345 women participated in this study. Complete specimens were available from 303 women. We excluded 65 neonates for prematurity and multiple pregnancies and analysed data from 238 mother–term neonate dyads (Figure 1). There were no exclusion criteria. Maternal and infant demographic data were extracted from the medical records once the infants were born. All data were entered into a Research Electronic Data Capture (RedCAP) database and de-identified for analysis.

### 2.2. Recruitment and Study Participants

All pregnant women were invited to be recruited when presenting to the hospital by research nurses and midwives. In this report we focused on singleton term mother–infant dyads only. Mother–infant dyads from premature (<37 completed weeks of GA) births and multiple pregnancies (twins and triplets) were excluded.

#### 2.2.1. Data on Maternal Smoking

A detailed maternal medical history was obtained during the first presentation to the antenatal clinics at TUH. Smoking status, as with a history of using other substances during pregnancy, was self-reported as per the hospital practice, and women had the option to omit the smoking information. A urine specimen for cotinine measurements was collected at the same time to determine more objectively if they had been exposed to nicotine during pregnancy. The collected samples were processed and stored at −80 °C, ready for analysis.

#### 2.2.2. Anthropometric Assessment of the Newborn

Infant anthropometric parameters (weight, length and head circumference) were measured after birth. Birth weight was measured using an electronic infant weighing scale (Seca 727 Electronic baby scale, Seca Deutschland, Hamburg, Germany) and were classified as small for gestational age (SGA; birth weight < 10th percentile for the gestational age (GA)) and appropriate for gestational age (AGA; birth weight being between 10th and 90th centile for GA) [17]. Gender was extracted from the infant medical records and birth weight Z-scores were calculated.

### 2.3. Maternal Urinary Cotinine Collection and Measurement

Urine was collected from women presenting for their antenatal visit. Urine cotinine is not normally detected in non-smokers unless they have been within an environment with much cigarette smoke (nicotine exposure via passive smoking) [15]. The Human Cotinine Eliza kit (ABNOVA, Taipei, Taiwan) had a detection limit of 1 ng/mL and a calibration range of 5 to 100 ng/mL. Simultaneous urine creatinine levels were measured to take account of variations in urinary dilution between individuals [18], and the maternal urine cotinine ratio was then calculated (UCCR) (ng/mmol). Urine cotinine concentrations below the assay’s < 5 ng/mL detection limit were recorded as 0 ng/mL (UCCR of 0 ng/mmol).

### 2.4. Statistical Analysis

The normality of the variables was determined by the D’Agostino–Pearson test [19]. The results are expressed as the means [standard deviation (S.D.)] for continuous, normally distributed data and as the median (interquartile range [IQR]) for continuous, non-normally distributed data. Comparisons of the means of normally distributed data were made using *t*-tests, and the Mann–Whitney test was used for non-normally distributed data. Differences in categorical variables were analysed by the Chi-squared test or Fisher’s exact test. Pearson correlation or Spearman’s rank-order correlation coefficient was used when appropriate. A *p* value < 0.05 was considered statistically significant. Statistical analyses were performed using MedCalc^®^ Statistical Software version 20.217 (MedCalc Software Ltd., Ostend, Belgium).

## 3. Results

### 3.1. Maternal Demographics and Urine Cotinine Analysis

From the whole cohort of pregnant women, 16% (38/238) self-reported as smokers compared to 50.4% (120/238) of the women showing cotinine in their urine sample (chi-square 39.7, *p* < 0.0001). The median UCCR was 0.07 [0.0–2.2] ng/mmol. The mean GA for the women when the urine was collected was 33.4 (5.7) weeks. There were no statistical differences between any maternal variables between self-reported smokers and non-smokers in terms of age, body mass index, mode of birth, history of alcohol consumption or recreational drugs recorded at the time of their antenatal check-up (refer to Table 1 for maternal demographics). The percentages of women with a diagnosis of eclampsia or diabetes mellitus or gestational diabetes were similar.

### 3.2. Cotinine Exposure on Anthropometric Measurements of the Newborn

The mean birth weight for the whole cohort was 3375 (498) g, and the mean GA was 38.9 (1.2) weeks. Table 2 compares neonates who were born to women without cotinine detected in their urine compared to those with cotinine.

**Figure 2:**
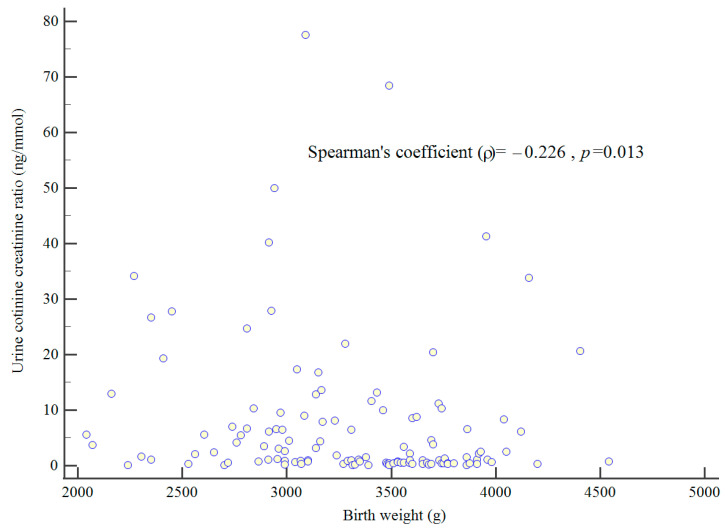
Correlation between birth weight and UCCR.

Neonates born to women with cotinine in their urine had significantly smaller birth weights (3293 (519) vs. 3458 (464) g, *p* = 0.010) and birth weight Z scores (−0.16 (1.04) vs. 0.17 (0.93), *p* = 0.010). There were also significantly more neonates born SGA to women with cotinine in their urine. There was a significant negative correlation between birth weight and UCCR (Spearman’s coefficient = −0.0226, *p* = 0.013) (Figure 2). Female babies born to women with nicotine in their urine were significantly smaller compared to those without nicotine in the urine (3121 (559) vs. 3449 (471); *p* = 0.001). However, there was no significant difference in the birth weights of male neonates born to women with nicotine in their urine compared to those without nicotine in their urine (3470 (459) vs. 3424 (448), *p* = 0.580).

## 4. Discussion

In our cohort, from regional Australia, 16% of women self-reported smoking during pregnancy at their antenatal check-up. These percentages are similar to 2021 statistics in inner regional (13.3%) and outer regional (15.5%) Australia [6], which is almost double the number of pregnant women reporting smoking at any time during pregnancy (8.7%) in Australia [5]. Of concern, 50.7% of the entire cohort had nicotine metabolites present in their urine (detected via urine cotinine levels), which is well above the national percentage, including very remote Australia (35.5%). These findings highlight a major discrepancy between reported smoking habits and true exposure. It is well established that nicotine-induced vasoconstriction and placental dysfunction can lead to a reduced birth weight in infants.

Birth weight is a key indicator of infant health and a crucial determinant of survival and well-being. Currently, routine antenatal checks rely on self-reporting of nicotine exposure, which may lead to missed opportunities to identify infants at high risk of adverse effects. Indeed, our findings show that higher levels of nicotine exposure during pregnancy (measured from urine cotinine levels and urine cotinine when adjusted for creatinine) markedly reduced the birth weight of the neonates, with more neonates being born small for gestational age compared to the neonates born to women that did not smoke during pregnancy (evidenced by absences of cotinine in urine).

Furthermore, female term neonates showed increased susceptibility to nicotine exposure, exhibiting significantly lower birth weights to mothers with cotinine in urine. Consequently, their vulnerability to developing well documented long-term adverse effects of being born small for gestational age is possibly increased [20]. Interestingly, this relationship was not evident in male neonates. It is important to note that, despite there being no difference between male and female neonates born to non-smokers in our cohort, we recognise that the apparent differences between male and female neonates born to smokers could just be a part of normal differences that exist between male and female neonates. A larger study is necessary to determine these sex differences. Nevertheless, previous studies have also shown similar sex trends. Voigt et al. [21] investigated the sex-specific risk of maternal smoking during pregnancy on the birth weight and the proportion of SGA newborns in 888,632 (49.9%) of 1,815,318 singleton births in Germany from 1995 to 1997 for whom data on maternal cigarette consumption were available. They reported that heavy smoking (>than 21 cigarettes/day) increased the risk of SGA 3.15-fold for a boy but 3.51-fold for a girl (*p* < 0.001) and concluded that, in heavy smokers, the negative effect of maternal smoking during pregnancy on the mean birth weight and risk of SGA is significantly more significant in female neonates. On the other hand, a population-based study of 23 African countries investigating the impact of environmental tobacco smoking (ETS) involving 208,027 newborns showed ETS had a more negative effect on male neonates than on female neonates [22]. These differences between studies could result from inaccuracies of smoking disclosure, as highlighted in our findings. It is imperative to improve the accuracy of smoking self-reporting by educating pregnant women on the importance of accurate reporting and considering incorporating biochemical verification by implementing non-invasive measurements of urine cotinine levels (as conducted in this study) as part of prenatal care to complement medical reporting.

UCCR remains a research tool and has not yet been recommended as part of routine clinical care by the professional bodies involved in determining clinical guidelines in obstetrics [23,24]. Cotinine assays have a low detection threshold; hence, it is possible that the actual number of women with detectable urine cotinine could be higher should a more sensitive assay become available. In this regard, a limitation of our study is that urine cotinine measurement has the inability to determine the nature of nicotine exposure during pregnancy, i.e., smoking (or using e-cigarettes), passive smoking, smoking cannabis linked with tobacco, or use of smokeless tobacco, whose increasing importance was highlighted recently in a large Swedish systematic review [25]. Another limitation of our study is that urine cotinine concentrations below the assay’s < 5 ng/mmol detection limit were recorded as 0 ng/mmol, further highlighting the importance of improving record-taking. Together, considering our findings, we have some strategic suggestions to help improve medical reporting on smoking habits. (1) Training healthcare providers in more effective communication, including making patients aware that smoking habits include other forms of smoking (including passive smoking of cigarettes and/or e-cigarettes, use of e-cigarettes, use of non-tobacco cigarettes). (2) Reflecting on biases and creating a more non-judgemental environment, whilst considering cultural sensitivity. (3) Using motivational interviewing techniques to encourage honest communication about smoking habits, which involve open-ended questions, reflective listening, and affirmations. (4) Exploring and implementing more private and less confrontational strategies, including using anonymous questionnaires and use of digital tools (apps that allow women to report smoking habits, remote access to increase routine check-ups, and with embedded educational information). (5) Introducing smoking cessation programs or re-evaluating current smoking cessation programs to better attract women residing in regional and remote regions.

As with many other clinical studies worldwide [26], our study was impacted significantly by the COVID-19 pandemic due to public health measures, which inadvertently resulted in barriers around requirements for in-person contact in enrolment and assessment for the families in our study. The main strength of this study was the large sample size of mother–neonate dyads that were analysed. The study utilised a non-invasive method to assess antenatal nicotine exposure, and the analysis was carried out by author (DR) who was blinded to the clinical information. The methods used in this study are relatively straightforward, and they could be carried out in a multicenter setting.

## 5. Conclusions

Urine cotinine was detected in half of the women in our cohort, and there was a significant negative correlation with birth weight. Neonates born to women with cotinine in their urine had significantly smaller birth weights, and significantly more neonates were born SGA. Whilst currently a research tool, the introduction of routine urine cotinine–creatinine ratio measurements in clinical practice might be beneficial to more accurately determine the impact of consuming nicotine during pregnancy on neonatal outcomes.

## Figures and Tables

**Table 1:** Comparison of maternal demography between smokers and non-smokers.

	Non-Smokers (N = 200)	Smokers (N = 38)	*p*-Value
Mean (SD) maternal age (years)	29.5 (6.0)	29.2 (6.3)	0.793
Mean (SD) maternal BMI (kg/mL)	28.8 (7.3)	27.0 (7.5)	0.274
Mode of birth (SVB/Other) (N)	122/78	22/16	0.591
History of alcohol consumption (%/N)	5.5 (11)	0	0.362
Other recreational drug use (%/N)	7.5 (15)	5.3 (2)	1.000
Maternal diagnosis of eclampsia (%/N)	5.5 (11)	5.3 (2)	1.000
Maternal diagnosis of DM/GDM (%/N)	31.5 (63)	34.2 (13)	0.851

Abbreviations: SD (standard deviation), BMI (body mass index), SVB (spontaneous vaginal birth), DM (diabetes mellitus), GDM (gestational diabetes mellitus), N (sample size).

**Table 2:** Comparison of neonates born to women without cotinine detected in their urine to those with cotinine.

Neonatal Variables	Maternal Urine Cotinine Not Detected	Maternal Urine Cotinine Detected	*p*
Birth weight (g (SD))	3458 (464)	3293 (519)	0.010
Birth weight Z score (SD)	0.17 (0.93)	−0.16 (1.04)	0.010
Head circumference (cm (SD))	34.9 (1.2)	34.7 (1.7)	0.204
Length (cm (SD))	50.4 (2.3)	49.8 (3.0)	0.074
Gestation (weeks (SD))	39.0 (1.1)	38.8 (2.0)	0.223
Female/Male (N)	64/54	52/68	0.093
SGA/AGA (N)	3/115	13/107	0.011

Abbreviation: N (number), SD (standard deviation), SGA (small for gestation age), AGA (appropriate for gestational age).

## Data Availability

Dataset available on request from the authors. The raw data supporting the conclusions of this article will be made available by the authors on request.
